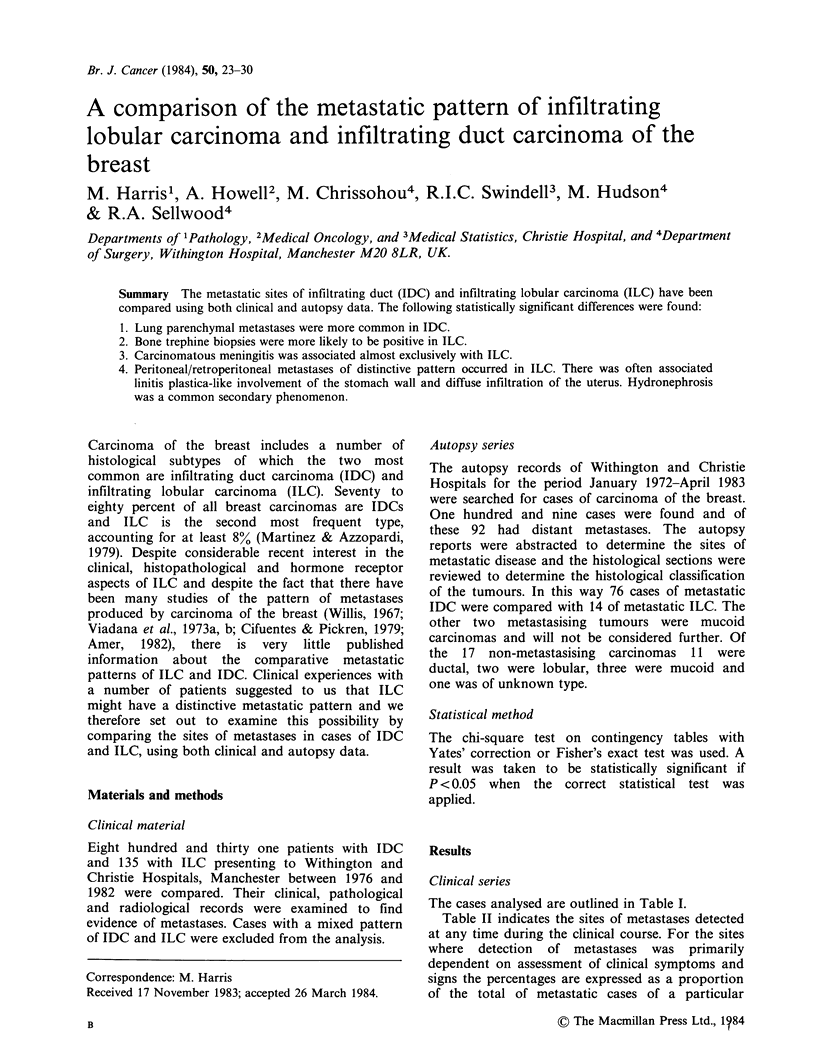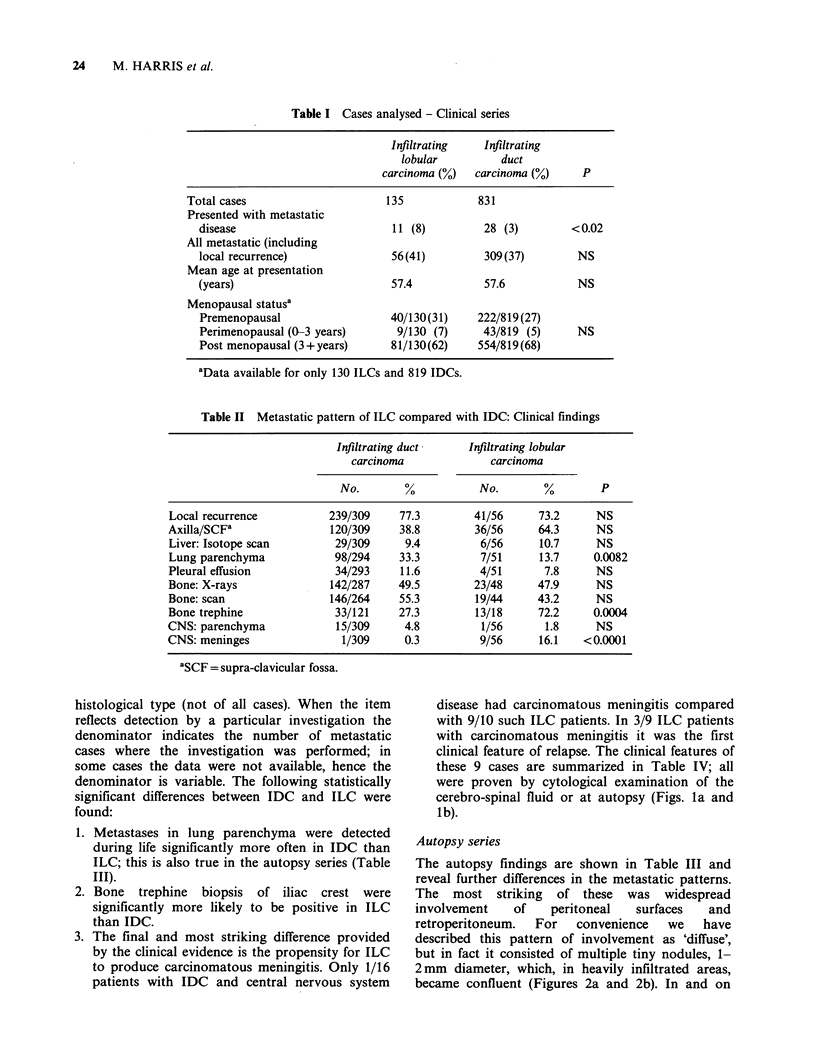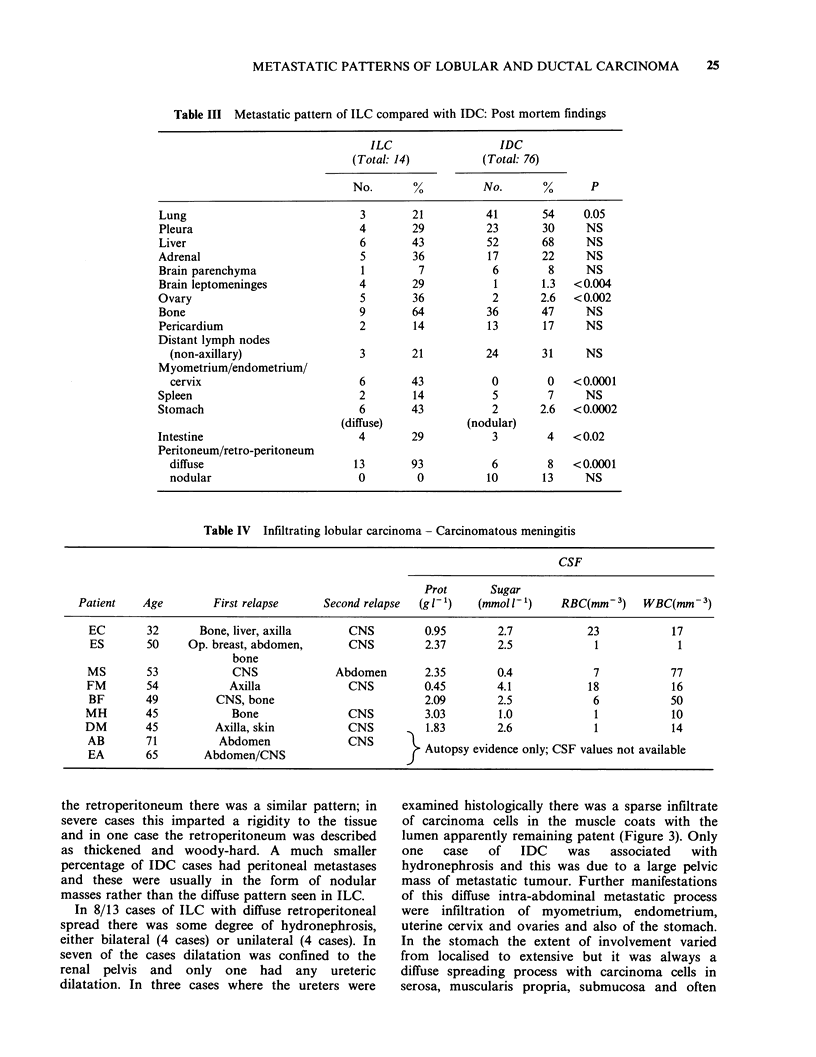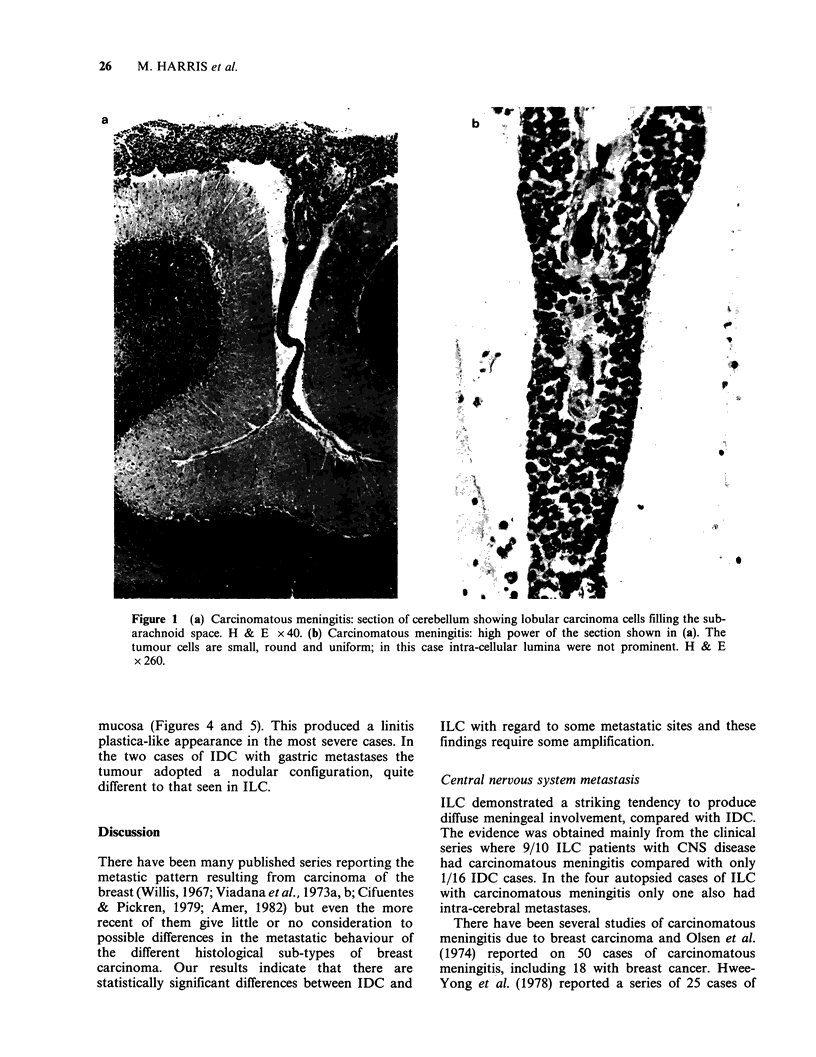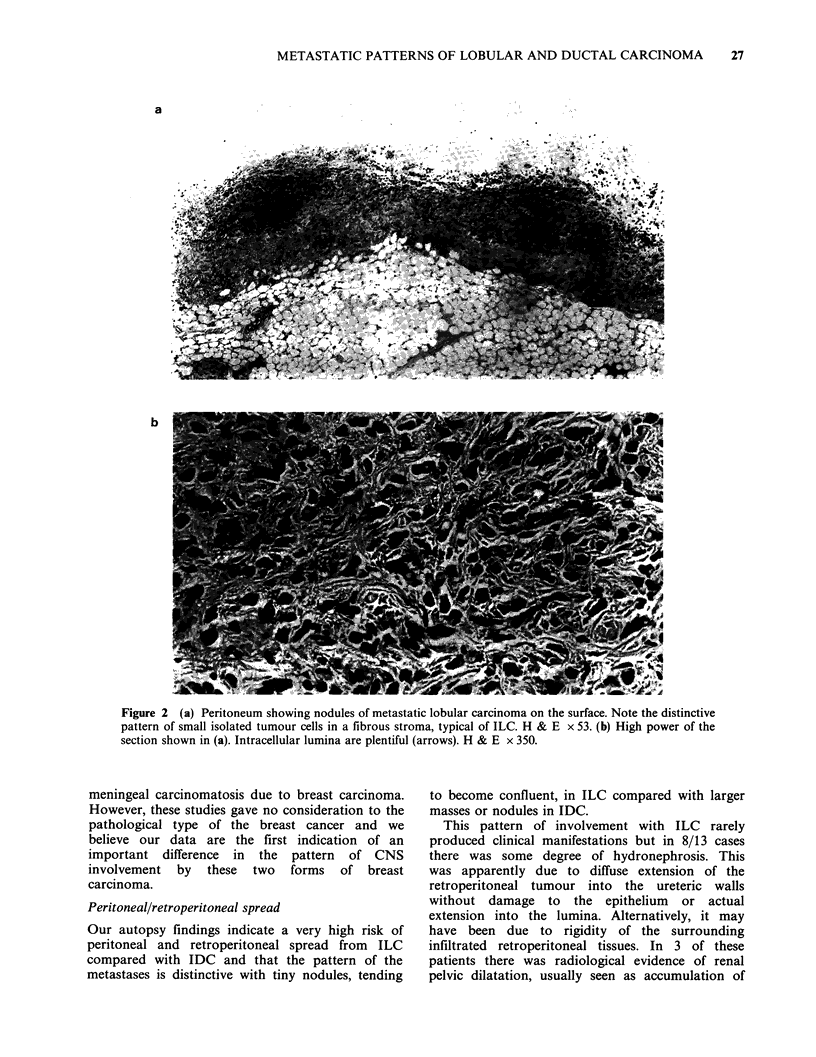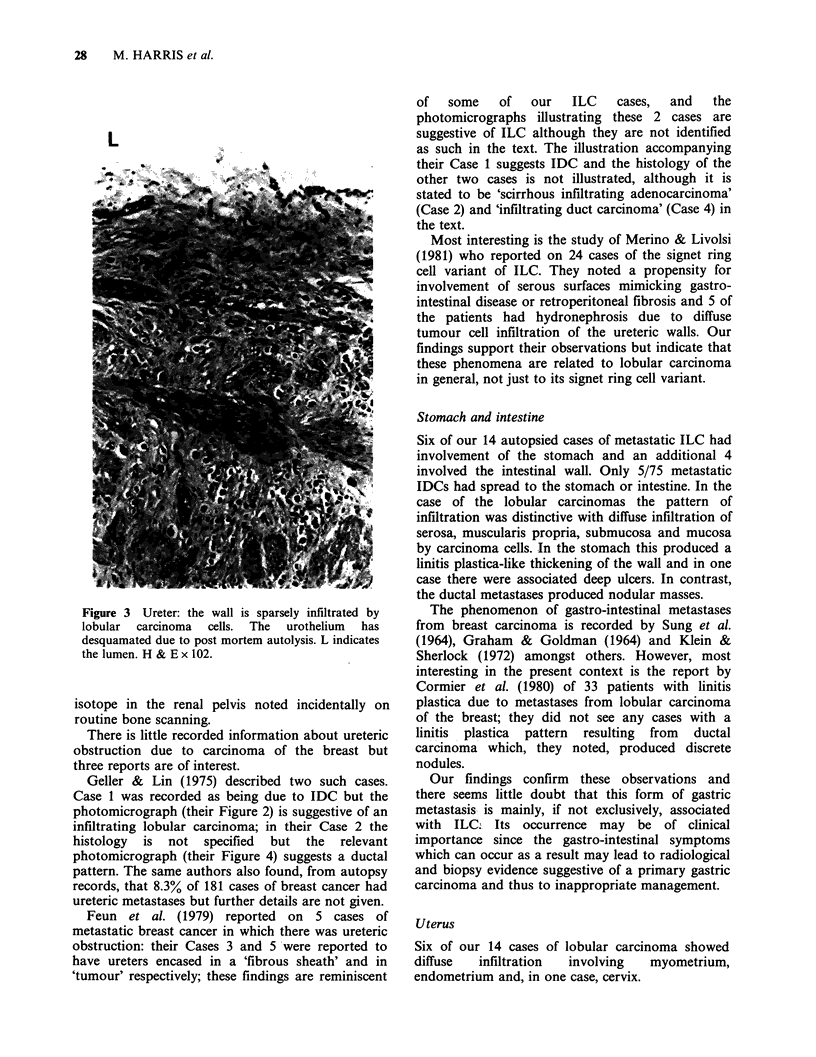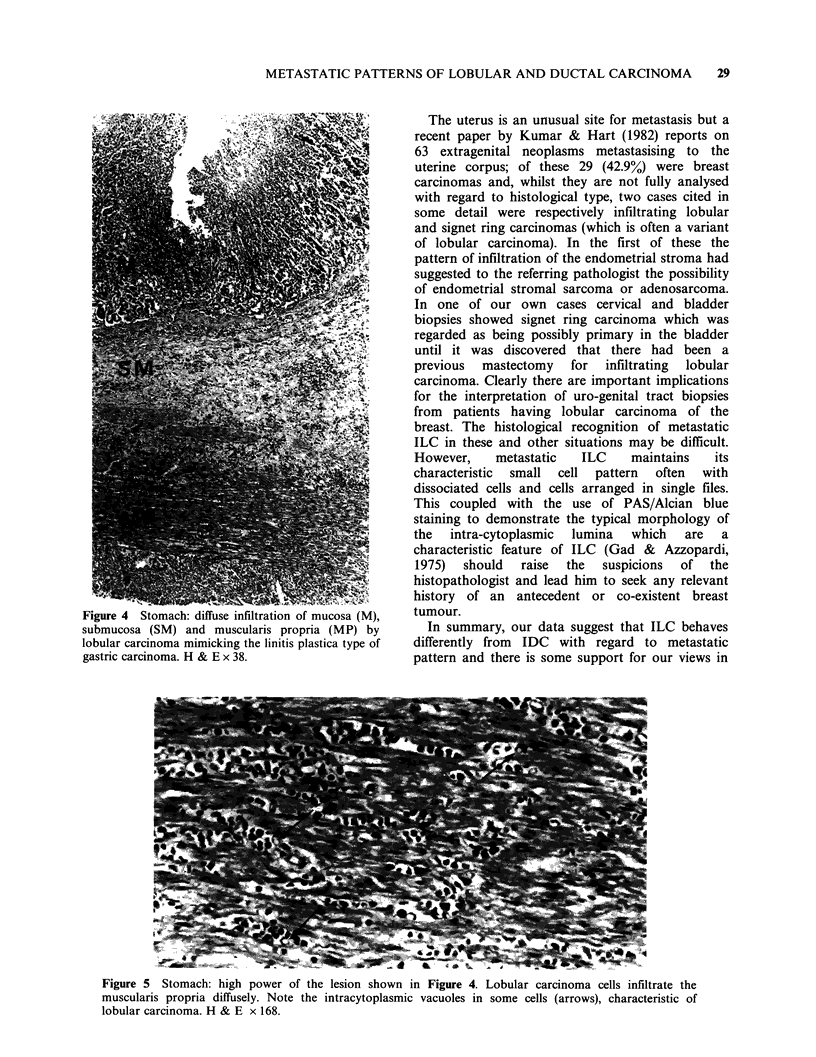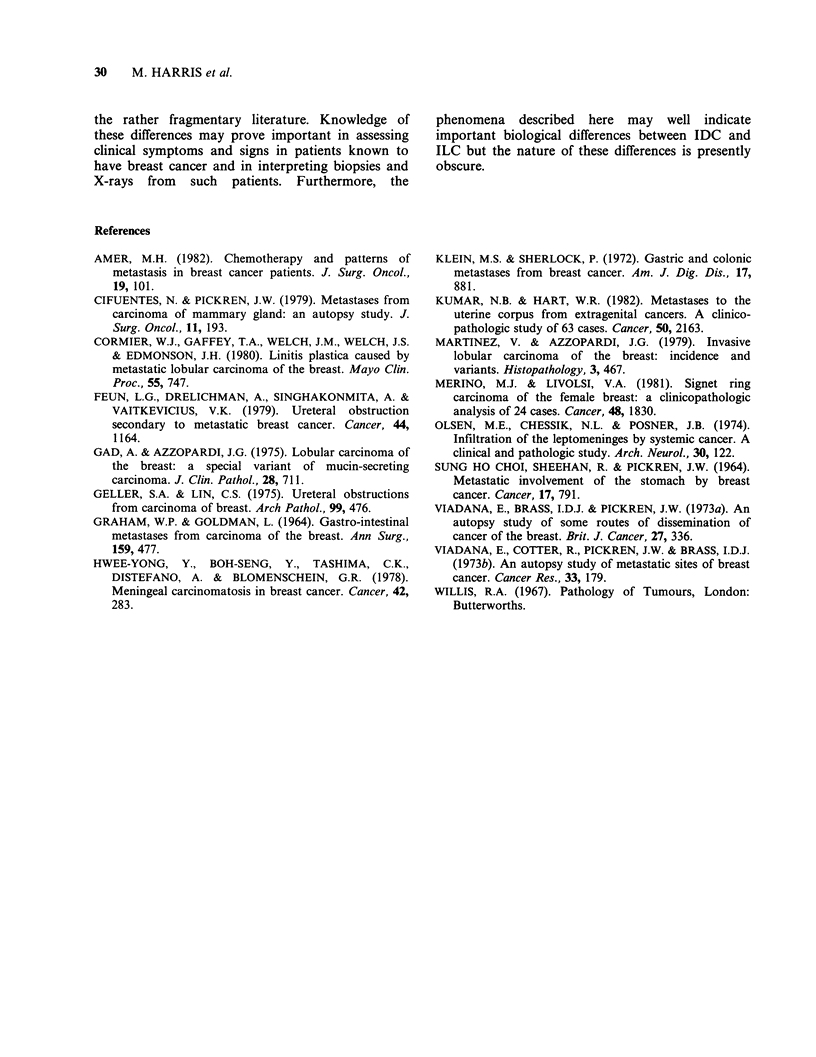# A comparison of the metastatic pattern of infiltrating lobular carcinoma and infiltrating duct carcinoma of the breast.

**DOI:** 10.1038/bjc.1984.135

**Published:** 1984-07

**Authors:** M. Harris, A. Howell, M. Chrissohou, R. I. Swindell, M. Hudson, R. A. Sellwood

## Abstract

**Images:**


*Br. J. Cancer* (1984), **50**, 23–30

### A comparison of the metastatic pattern of infiltrating lobular carcinoma and infiltrating duct carcinoma of the breast

M. Harris[1], A. Howell[2], M. Chrissohou[4], R.I.C. Swindell[3], M. Hudson[4] & R.A. Sellwood[4]

*Departments of [1]Pathology, [2]Medical Oncology, and [3]Medical Statistics, Christie Hospital, and [4]Department of Surgery, Withington Hospital, Manchester M20 8LR, UK.*

**Summary** The metastatic sites of infiltrating duct (IDC) and infiltrating lobular carcinoma (ILC) have been compared using both clinical and autopsy data. The following statistically significant differences were found:

1. Lung parenchymal metastases were more common in IDC.
2. Bone trephine biopsies were more likely to be positive in ILC.
3. Carcinomatous meningitis was associated almost exclusively with ILC.
4. Peritoneal/retroperitoneal metastases of distinctive pattern occurred in ILC. There was often associated linitis plastica-like involvement of the stomach wall and diffuse infiltration of the uterus. Hydronephrosis was a common secondary phenomenon.

Carcinoma of the breast includes a number of histological subtypes of which the two most common are infiltrating duct carcinoma (IDC) and infiltrating lobular carcinoma (ILC). Seventy to eighty percent of all breast carcinomas are IDCs and ILC is the second most frequent type, accounting for at least 8% (Martinez & Azzopardi, 1979). Despite considerable recent interest in the clinical, histopathological and hormone receptor aspects of ILC and despite the fact that there have been many studies of the pattern of metastases produced by carcinoma of the breast (Willis, 1967; Viadana *et al.*, 1973a, b; Cifuentes & Pickren, 1979; Amer, 1982), there is very little published information about the comparative metastatic patterns of ILC and IDC. Clinical experiences with a number of patients suggested to us that ILC might have a distinctive metastatic pattern and we therefore set out to examine this possibility by comparing the sites of metastases in cases of IDC and ILC, using both clinical and autopsy data.

### Materials and methods

### *Clinical material*

Eight hundred and thirty one patients with IDC and 135 with ILC presenting to Withington and Christie Hospitals, Manchester between 1976 and 1982 were compared. Their clinical, pathological and radiological records were examined to find evidence of metastases. Cases with a mixed pattern of IDC and ILC were excluded from the analysis.

Correspondence: M. Harris
Received 17 November 1983; accepted 26 March 1984.

### *Autopsy series*

The autopsy records of Withington and Christie Hospitals for the period January 1972–April 1983 were searched for cases of carcinoma of the breast. One hundred and nine cases were found and of these 92 had distant metastases. The autopsy reports were abstracted to determine the sites of metastatic disease and the histological sections were reviewed to determine the histological classification of the tumours. In this way 76 cases of metastatic IDC were compared with 14 of metastatic ILC. The other two metastasising tumours were mucoid carcinomas and will not be considered further. Of the 17 non-metastasising carcinomas 11 were ductal, two were lobular, three were mucoid and one was of unknown type.

### *Statistical method*

The chi-square test on contingency tables with Yates' correction or Fisher's exact test was used. A result was taken to be statistically significant if $P<0.05$ when the correct statistical test was applied.

### Results

### *Clinical series*

The cases analysed are outlined in Table I.

Table II indicates the sites of metastases detected at any time during the clinical course. For the sites where detection of metastases was primarily dependent on assessment of clinical symptoms and signs the percentages are expressed as a proportion of the total of metastatic cases of a particular

© The Macmillan Press Ltd., 1984

**Table I** Cases analysed – Clinical series

| | *Infiltrating lobular carcinoma* (%) | *Infiltrating duct carcinoma* (%) | *P* |
|---|---|---|---|
| Total cases | 135 | 831 | |
| Presented with metastatic disease | 11 (8) | 28 (3) | <0.02 |
| All metastatic (including local recurrence) | 56(41) | 309(37) | NS |
| Mean age at presentation (years) | 57.4 | 57.6 | NS |
| Menopausal status[a] | | | |
| Premenopausal | 40/130(31) | 222/819(27) | |
| Perimenopausal (0–3 years) | 9/130 (7) | 43/819 (5) | NS |
| Post menopausal (3+years) | 81/130(62) | 554/819(68) | |

[a]Data available for only 130 ILCs and 819 IDCs.

**Table II** Metastatic pattern of ILC compared with IDC: Clinical findings

| | *Infiltrating duct carcinoma* | | *Infiltrating lobular carcinoma* | | |
|---|---|---|---|---|---|
| | *No.* | % | *No.* | % | *P* |
| Local recurrence | 239/309 | 77.3 | 41/56 | 73.2 | NS |
| Axilla/SCF[a] | 120/309 | 38.8 | 36/56 | 64.3 | NS |
| Liver: Isotope scan | 29/309 | 9.4 | 6/56 | 10.7 | NS |
| Lung parenchyma | 98/294 | 33.3 | 7/51 | 13.7 | 0.0082 |
| Pleural effusion | 34/293 | 11.6 | 4/51 | 7.8 | NS |
| Bone: X-rays | 142/287 | 49.5 | 23/48 | 47.9 | NS |
| Bone: scan | 146/264 | 55.3 | 19/44 | 43.2 | NS |
| Bone trephine | 33/121 | 27.3 | 13/18 | 72.2 | 0.0004 |
| CNS: parenchyma | 15/309 | 4.8 | 1/56 | 1.8 | NS |
| CNS: meninges | 1/309 | 0.3 | 9/56 | 16.1 | <0.0001 |

[a]SCF = supra-clavicular fossa.

histological type (not of all cases). When the item reflects detection by a particular investigation the denominator indicates the number of metastatic cases where the investigation was performed; in some cases the data were not available, hence the denominator is variable. The following statistically significant differences between IDC and ILC were found:

1. Metastases in lung parenchyma were detected during life significantly more often in IDC than ILC; this is also true in the autopsy series (Table III).
2. Bone trephine biopsis of iliac crest were significantly more likely to be positive in ILC than IDC.
3. The final and most striking difference provided by the clinical evidence is the propensity for ILC to produce carcinomatous meningitis. Only 1/16 patients with IDC and central nervous system disease had carcinomatous meningitis compared with 9/10 such ILC patients. In 3/9 ILC patients with carcinomatous meningitis it was the first clinical feature of relapse. The clinical features of these 9 cases are summarized in Table IV; all were proven by cytological examination of the cerebro-spinal fluid or at autopsy (Figs. 1a and 1b).

*Autopsy series*

The autopsy findings are shown in Table III and reveal further differences in the metastatic patterns. The most striking of these was widespread involvement of peritoneal surfaces and retroperitoneum. For convenience we have described this pattern of involvement as 'diffuse', but in fact it consisted of multiple tiny nodules, 1–2 mm diameter, which, in heavily infiltrated areas, became confluent (Figures 2a and 2b). In and on

**Table III** Metastatic pattern of ILC compared with IDC: Post mortem findings

| | *ILC* (*Total: 14*) | | *IDC* (*Total: 76*) | | |
|---|---|---|---|---|---|
| | No. | % | *No.* | % | *P* |
| Lung | 3 | 21 | 41 | 54 | 0.05 |
| Pleura | 4 | 29 | 23 | 30 | NS |
| Liver | 6 | 43 | 52 | 68 | NS |
| Adrenal | 5 | 36 | 17 | 22 | NS |
| Brain parenchyma | 1 | 7 | 6 | 8 | NS |
| Brain leptomeninges | 4 | 29 | 1 | 1.3 | <0.004 |
| Ovary | 5 | 36 | 2 | 2.6 | <0.002 |
| Bone | 9 | 64 | 36 | 47 | NS |
| Pericardium | 2 | 14 | 13 | 17 | NS |
| Distant lymph nodes (non-axillary) | 3 | 21 | 24 | 31 | NS |
| Myometrium/endometrium/ cervix | 6 | 43 | 0 | 0 | <0.0001 |
| Spleen | 2 | 14 | 5 | 7 | NS |
| Stomach | 6 (diffuse) | 43 | 2 (nodular) | 2.6 | <0.0002 |
| Intestine | 4 | 29 | 3 | 4 | <0.02 |
| Peritoneum/retro-peritoneum | | | | | |
| diffuse | 13 | 93 | 6 | 8 | <0.0001 |
| nodular | 0 | 0 | 10 | 13 | NS |

**Table IV** Infiltrating lobular carcinoma – Carcinomatous meningitis

| | | | | *CSF* | | | |
|---|---|---|---|---|---|---|---|
| *Patient* | *Age* | *First relapse* | *Second relapse* | *Prot* ($g\,l^{-1}$) | *Sugar* ($mmol\,l^{-1}$) | *RBC*($mm^{-3}$) | *WBC*($mm^{-3}$) |
| EC | 32 | Bone, liver, axilla | CNS | 0.95 | 2.7 | 23 | 17 |
| ES | 50 | Op. breast, abdomen, bone | CNS | 2.37 | 2.5 | 1 | 1 |
| MS | 53 | CNS | Abdomen | 2.35 | 0.4 | 7 | 77 |
| FM | 54 | Axilla | CNS | 0.45 | 4.1 | 18 | 16 |
| BF | 49 | CNS, bone | | 2.09 | 2.5 | 6 | 50 |
| MH | 45 | Bone | CNS | 3.03 | 1.0 | 1 | 10 |
| DM | 45 | Axilla, skin | CNS | 1.83 | 2.6 | 1 | 14 |
| AB | 71 | Abdomen | CNS | } Autopsy evidence only; CSF values not available | | | |
| EA | 65 | Abdomen/CNS | | | | | |

the retroperitoneum there was a similar pattern; in severe cases this imparted a rigidity to the tissue and in one case the retroperitoneum was described as thickened and woody-hard. A much smaller percentage of IDC cases had peritoneal metastases and these were usually in the form of nodular masses rather than the diffuse pattern seen in ILC.

In 8/13 cases of ILC with diffuse retroperitoneal spread there was some degree of hydronephrosis, either bilateral (4 cases) or unilateral (4 cases). In seven of the cases dilatation was confined to the renal pelvis and only one had any ureteric dilatation. In three cases where the ureters were examined histologically there was a sparse infiltrate of carcinoma cells in the muscle coats with the lumen apparently remaining patent (Figure 3). Only one case of IDC was associated with hydronephrosis and this was due to a large pelvic mass of metastatic tumour. Further manifestations of this diffuse intra-abdominal metastatic process were infiltration of myometrium, endometrium, uterine cervix and ovaries and also of the stomach. In the stomach the extent of involvement varied from localised to extensive but it was always a diffuse spreading process with carcinoma cells in serosa, muscularis propria, submucosa and often

**Figure 1** (**a**) Carcinomatous meningitis: section of cerebellum showing lobular carcinoma cells filling the sub-arachnoid space. H & E ×40. (**b**) Carcinomatous meningitis: high power of the section shown in (**a**). The tumour cells are small, round and uniform; in this case intra-cellular lumina were not prominent. H & E ×260.

mucosa (Figures 4 and 5). This produced a linitis plastica-like appearance in the most severe cases. In the two cases of IDC with gastric metastases the tumour adopted a nodular configuration, quite different to that seen in ILC.

### Discussion

There have been many published series reporting the metastic pattern resulting from carcinoma of the breast (Willis, 1967; Viadana *et al.*, 1973a, b; Cifuentes & Pickren, 1979; Amer, 1982) but even the more recent of them give little or no consideration to possible differences in the metastatic behaviour of the different histological sub-types of breast carcinoma. Our results indicate that there are statistically significant differences between IDC and ILC with regard to some metastatic sites and these findings require some amplification.

### *Central nervous system metastasis*

ILC demonstrated a striking tendency to produce diffuse meningeal involvement, compared with IDC. The evidence was obtained mainly from the clinical series where 9/10 ILC patients with CNS disease had carcinomatous meningitis compared with only 1/16 IDC cases. In the four autopsied cases of ILC with carcinomatous meningitis only one also had intra-cerebral metastases.

There have been several studies of carcinomatous meningitis due to breast carcinoma and Olsen *et al.* (1974) reported on 50 cases of carcinomatous meningitis, including 18 with breast cancer. Hwee-Yong *et al.* (1978) reported a series of 25 cases of

**Figure 2** (**a**) Peritoneum showing nodules of metastatic lobular carcinoma on the surface. Note the distinctive pattern of small isolated tumour cells in a fibrous stroma, typical of ILC. H & E ×53. (**b**) High power of the section shown in (**a**). Intracellular lumina are plentiful (arrows). H & E ×350.

meningeal carcinomatosis due to breast carcinoma. However, these studies gave no consideration to the pathological type of the breast cancer and we believe our data are the first indication of an important difference in the pattern of CNS involvement by these two forms of breast carcinoma.

*Peritoneal/retroperitoneal spread*

Our autopsy findings indicate a very high risk of peritoneal and retroperitoneal spread from ILC compared with IDC and that the pattern of the metastases is distinctive with tiny nodules, tending to become confluent, in ILC compared with larger masses or nodules in IDC.

This pattern of involvement with ILC rarely produced clinical manifestations but in 8/13 cases there was some degree of hydronephrosis. This was apparently due to diffuse extension of the retroperitoneal tumour into the ureteric walls without damage to the epithelium or actual extension into the lumina. Alternatively, it may have been due to rigidity of the surrounding infiltrated retroperitoneal tissues. In 3 of these patients there was radiological evidence of renal pelvic dilatation, usually seen as accumulation of

**Figure 3** Ureter: the wall is sparsely infiltrated by lobular carcinoma cells. The urothelium has desquamated due to post mortem autolysis. L indicates the lumen. H & E × 102.

isotope in the renal pelvis noted incidentally on routine bone scanning.

There is little recorded information about ureteric obstruction due to carcinoma of the breast but three reports are of interest.

Geller & Lin (1975) described two such cases. Case 1 was recorded as being due to IDC but the photomicrograph (their Figure 2) is suggestive of an infiltrating lobular carcinoma; in their Case 2 the histology is not specified but the relevant photomicrograph (their Figure 4) suggests a ductal pattern. The same authors also found, from autopsy records, that 8.3% of 181 cases of breast cancer had ureteric metastases but further details are not given.

Feun *et al.* (1979) reported on 5 cases of metastatic breast cancer in which there was ureteric obstruction: their Cases 3 and 5 were reported to have ureters encased in a 'fibrous sheath' and in 'tumour' respectively; these findings are reminiscent of some of our ILC cases, and the photomicrographs illustrating these 2 cases are suggestive of ILC although they are not identified as such in the text. The illustration accompanying their Case 1 suggests IDC and the histology of the other two cases is not illustrated, although it is stated to be 'scirrhous infiltrating adenocarcinoma' (Case 2) and 'infiltrating duct carcinoma' (Case 4) in the text.

Most interesting is the study of Merino & Livolsi (1981) who reported on 24 cases of the signet ring cell variant of ILC. They noted a propensity for involvement of serous surfaces mimicking gastro-intestinal disease or retroperitoneal fibrosis and 5 of the patients had hydronephrosis due to diffuse tumour cell infiltration of the ureteric walls. Our findings support their observations but indicate that these phenomena are related to lobular carcinoma in general, not just to its signet ring cell variant.

### *Stomach and intestine*

Six of our 14 autopsied cases of metastatic ILC had involvement of the stomach and an additional 4 involved the intestinal wall. Only 5/75 metastatic IDCs had spread to the stomach or intestine. In the case of the lobular carcinomas the pattern of infiltration was distinctive with diffuse infiltration of serosa, muscularis propria, submucosa and mucosa by carcinoma cells. In the stomach this produced a linitis plastica-like thickening of the wall and in one case there were associated deep ulcers. In contrast, the ductal metastases produced nodular masses.

The phenomenon of gastro-intestinal metastases from breast carcinoma is recorded by Sung *et al.* (1964), Graham & Goldman (1964) and Klein & Sherlock (1972) amongst others. However, most interesting in the present context is the report by Cormier *et al.* (1980) of 33 patients with linitis plastica due to metastases from lobular carcinoma of the breast; they did not see any cases with a linitis plastica pattern resulting from ductal carcinoma which, they noted, produced discrete nodules.

Our findings confirm these observations and there seems little doubt that this form of gastric metastasis is mainly, if not exclusively, associated with ILC. Its occurrence may be of clinical importance since the gastro-intestinal symptoms which can occur as a result may lead to radiological and biopsy evidence suggestive of a primary gastric carcinoma and thus to inappropriate management.

### *Uterus*

Six of our 14 cases of lobular carcinoma showed diffuse infiltration involving myometrium, endometrium and, in one case, cervix.

**Figure 4** Stomach: diffuse infiltration of mucosa (M), submucosa (SM) and muscularis propria (MP) by lobular carcinoma mimicking the linitis plastica type of gastric carcinoma. H & E × 38.

The uterus is an unusual site for metastasis but a recent paper by Kumar & Hart (1982) reports on 63 extragenital neoplasms metastasising to the uterine corpus; of these 29 (42.9%) were breast carcinomas and, whilst they are not fully analysed with regard to histological type, two cases cited in some detail were respectively infiltrating lobular and signet ring carcinomas (which is often a variant of lobular carcinoma). In the first of these the pattern of infiltration of the endometrial stroma had suggested to the referring pathologist the possibility of endometrial stromal sarcoma or adenosarcoma. In one of our own cases cervical and bladder biopsies showed signet ring carcinoma which was regarded as being possibly primary in the bladder until it was discovered that there had been a previous mastectomy for infiltrating lobular carcinoma. Clearly there are important implications for the interpretation of uro-genital tract biopsies from patients having lobular carcinoma of the breast. The histological recognition of metastatic ILC in these and other situations may be difficult. However, metastatic ILC maintains its characteristic small cell pattern often with dissociated cells and cells arranged in single files. This coupled with the use of PAS/Alcian blue staining to demonstrate the typical morphology of the intra-cytoplasmic lumina which are a characteristic feature of ILC (Gad & Azzopardi, 1975) should raise the suspicions of the histopathologist and lead him to seek any relevant history of an antecedent or co-existent breast tumour.

In summary, our data suggest that ILC behaves differently from IDC with regard to metastatic pattern and there is some support for our views in

**Figure 5** Stomach: high power of the lesion shown in **Figure** 4. Lobular carcinoma cells infiltrate the muscularis propria diffusely. Note the intracytoplasmic vacuoles in some cells (arrows), characteristic of lobular carcinoma. H & E × 168.

the rather fragmentary literature. Knowledge of these differences may prove important in assessing clinical symptoms and signs in patients known to have breast cancer and in interpreting biopsies and X-rays from such patients. Furthermore, the phenomena described here may well indicate important biological differences between IDC and ILC but the nature of these differences is presently obscure.

### References

AMER, M.H. (1982). Chemotherapy and patterns of metastasis in breast cancer patients. *J. Surg. Oncol.*, **19**, 101.

CIFUENTES, N. & PICKREN, J.W. (1979). Metastases from carcinoma of mammary gland: an autopsy study. *J. Surg. Oncol.*, **11**, 193.

CORMIER, W.J., GAFFEY, T.A., WELCH, J.M., WELCH, J.S. & EDMONSON, J.H. (1980). Linitis plastica caused by metastatic lobular carcinoma of the breast. *Mayo Clin. Proc.*, **55**, 747.

FEUN, L.G., DRELICHMAN, A., SINGHAKONMITA, A. & VAITKEVICIUS, V.K. (1979). Ureteral obstruction secondary to metastatic breast cancer. *Cancer*, **44**, 1164.

GAD, A. & AZZOPARDI, J.G. (1975). Lobular carcinoma of the breast: a special variant of mucin-secreting carcinoma. *J. Clin. Pathol.*, **28**, 711.

GELLER, S.A. & LIN, C.S. (1975). Ureteral obstructions from carcinoma of breast. *Arch Pathol.*, **99**, 476.

GRAHAM, W.P. & GOLDMAN, L. (1964). Gastro-intestinal metastases from carcinoma of the breast. *Ann Surg.*, **159**, 477.

HWEE-YONG, Y., BOH-SENG, Y., TASHIMA, C.K., DISTEFANO, A. & BLOMENSCHEIN, G.R. (1978). Meningeal carcinomatosis in breast cancer. *Cancer*, **42**, 283.

KLEIN, M.S. & SHERLOCK, P. (1972). Gastric and colonic metastases from breast cancer. *Am. J. Dig. Dis.*, **17**, 881.

KUMAR, N.B. & HART, W.R. (1982). Metastases to the uterine corpus from extragenital cancers. A clinicopathologic study of 63 cases. *Cancer*, **50**, 2163.

MARTINEZ, V. & AZZOPARDI, J.G. (1979). Invasive lobular carcinoma of the breast: incidence and variants. *Histopathology*, **3**, 467.

MERINO, M.J. & LIVOLSI, V.A. (1981). Signet ring carcinoma of the female breast: a clinicopathologic analysis of 24 cases. *Cancer*, **48**, 1830.

OLSEN, M.E., CHESSIK, N.L. & POSNER, J.B. (1974). Infiltration of the leptomeninges by systemic cancer. A clinical and pathologic study. *Arch. Neurol.*, **30**, 122.

SUNG HO CHOI, SHEEHAN, R. & PICKREN, J.W. (1964). Metastatic involvement of the stomach by breast cancer. *Cancer*, **17**, 791.

VIADANA, E., BRASS, I.D.J. & PICKREN, J.W. (1973*a*). An autopsy study of some routes of dissemination of cancer of the breast. *Brit. J. Cancer*, **27**, 336.

VIADANA, E., COTTER, R., PICKREN, J.W. & BRASS, I.D.J. (1973*b*). An autopsy study of metastatic sites of breast cancer. *Cancer Res.*, **33**, 179.

WILLIS, R.A. (1967). Pathology of Tumours, London: Butterworths.